# Adsorption of Cs Ions in Hydroxy-Al Interlayered Clay Minerals and the Aging Mechanism

**DOI:** 10.1021/acs.langmuir.5c05291

**Published:** 2026-02-21

**Authors:** Hiroshi Sakuma, Kenji Tamura, Shigeru Suehara, Kenjiro Hashi

**Affiliations:** † 52747 National Institute for Materials Science, 1-1 Namiki, Tsukuba, Ibaraki 305-0044, Japan; ‡ 52747 National Institute for Materials Science, 3-13 Sakura, Tsukuba, Ibaraki 305-0003, Japan

## Abstract

A decrease in the desorption rate of Cs^+^ from natural sediment was observed with increasing Cs^+^ sorption time. This aging effect poses a serious issue as it hinders the removal of radioactive cesium ions from natural sediments. In this study, adsorption and desorption experiments and molecular simulations were conducted on artificially weathered hydroxy-Al-interlayered clay minerals to elucidate the mechanism underlying this aging effect. The adsorption selectivity of Cs^+^ was independent of the hydroxy-Al concentration; however, the desorption rate from the low-concentration hydroxy-Al phlogopite was significantly lower than that from the high-concentration samples. This difference can be attributed to the presence of collapsed and wedge zones in the interlayer of low-concentration hydroxy-Al. During aging tests for Cs adsorption, a 5-fold coordination of Al was observed in its nuclear magnetic resonance spectrum. Molecular dynamics simulations revealed that Cs^+^ in the wedge zone was highly mobile owing to its weak interactions with the basal plane. Cs^+^ fixation was observed near the edges of the electrically neutral hydroxy-Al sheets, within the collapsed zone, and within the hydroxy-Al sheets. The proposed aging mechanism of Cs^+^ in hydroxy-Al-interlayered clay minerals involves two steps: (1) Cs^+^ penetrates the interlayer space, which is expanded by the presence of hydroxy-Al segments, and (2a) it gradually migrates into the collapsed zone or (2b) into the inner hydroxy-Al layers. The structure of the (2b) model can explain the presence of 5-fold coordination of Al, and the stability of Cs^+^ in the hydroxy-Al sheets was evaluated using density functional theory calculations. These findings can contribute to the development of an efficient desorption method for Cs^+^ from natural sediments and the design of materials capable of removing or immobilizing Cs^+^ from aqueous solutions.

## Graphical abstract

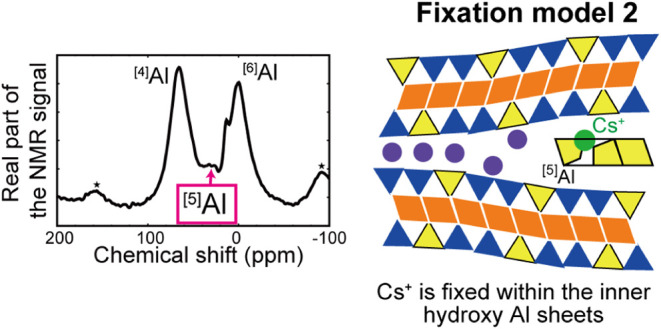

## Introduction

Following the 2011 Great East Japan Earthquake off the Pacific coast of To̅hoku and the subsequent tsunami, the accident at the Fukushima Daiichi Nuclear Power Plant released radioactive cesium, which spread widely across eastern Japan. The half-life of ^137^Cs is 30.2 years, leading to harmful long-term effects on the environment. One of the major challenges in the removal of radioactive cesium ions from sediments is their strong adsorption onto clay minerals, which makes desorption of Cs from clay minerals difficult.^1^ In addition, a decrease in the desorption rate was observed with increasing Cs sorption time (aging effect).^2,3^ To reveal the adsorption mechanism and identify adsorption sites, it is crucial to develop both efficient methods for Cs removal from sediments and materials capable of ensuring long-term Cs fixation.

Adsorption sites of Cs^+^ in clay minerals can be classified as follows: the external basal plane, interlayer space, edge plane, frayed-edge site (FES), and wedge zone near the hydroxy-interlayer in the interlayer space.^4–6^ The presence of highly selective sites for Cs^+^ relative to K^+^ in illite has been recognized, and these sites are thermodynamically reversible.^7^ The free energy for the exchange of K^+^ on the Cs^+^ at these high-selectivity sites is −23.5 kJ/mol, which is comparable to the simulated ion exchange energy of −23 kJ/mol for the exchange of Cs^+^ against K^+^ at a modeled FES.^8^ FESs can be characterized by the wedge interlayer space formed by the weathering of edge sites, and a slightly opened interlayer space is optimal for the adsorption of relatively large Cs^+^.^9^ However, these sites are thermodynamically reversible and do not explain the fixation of radioactive Cs^+^ over time.

The area near the Fukushima Daiichi nuclear power plant is covered mainly with weathered granitic soil. In the soil, weathered biotite (WB) is abundant, and radioactive Cs is adsorbed strongly on this WB.^3^ The fixation of radioactive ^137^Cs^+^ traces on biotite varies with artificial weathering.^10^ The distribution coefficients, *K*_d_, which are defined as the adsorbed ^137^Cs^+^ concentration on minerals divided by the concentration in the liquid phase, were similar among biotite, vermiculite, and hydroxy-Al interlayered vermiculite (HIV). However, the net ^137^Cs^+^ retention after desorption treatments by ion exchangers for vermiculite was higher than that for biotite and HIV. The strong retention of ^137^Cs^+^ by vermiculite may be explained by the collapse of the interlayer spaces in the ^137^Cs^+^ adsorbed FES or wedge zones. In contrast, hydroxy-Al inhibits the collapse of layers,^11^ which enables the desorption of ^137^Cs^+^ from HIV. We hypothesized that the presence of wedge zones and the collapse of the interlayer at the wedge zones may be essential for the retention of ^137^Cs^+^. It should be noted that the HIV used in their experiments inhibited the collapse of the layers; however, common HIV can cause partially collapsed interlayers. HIV forms a perfect solid solution between the end members of vermiculite and aluminous chlorite, and the number and density of hydroxy-Al vary depending on the formation conditions.^11^

The number of wedge zones in HIV depends on the number and size of the hydroxy-Al atoms in the layers. The radioactive cesium interception potential (RIP) has been used to estimate the capacity of the Cs-selectivity sites in wedge zones.^12^ The RIP depends on the number of hydroxy-Al atoms and has a maximum at 2–10 g Al/kg clay.^13^ Therefore, the behavior of Cs^+^ adsorption may be altered by the number of hydroxy-Al atoms in the layers. The *K*_d_ value of Cs adsorption in altered Al-phlogopite is higher at low Cs^+^ concentrations (<7.5 × 10^–4^ mmol L^–1^ Cs^+^), comparable to those found in actual contaminated soils, than in Na-, K-, Ca-, and Mg-phlogopites.^14^ This suggests that Cs^+^ decontamination from the environment could be facilitated by controlling the amount of hydroxy-Al in the layers.

In this study, the influence of the wedge and collapse zones in HIV on the adsorption and desorption of Cs^+^ was revealed by experiments and molecular simulations. Partially collapsed HIVs were experimentally modeled using artificially weathered phlogopite. Phlogopite is an endmember of biotite; therefore, this is an ideal material to study the Cs adsorption behavior. In addition, nuclear magnetic resonance (NMR) spectroscopy analysis was conducted due to the lack of Fe ions in phlogopite. In our previous studies, the synthesis of artificially altered phlogopite was achieved by the hydrothermal treatment of phlogopite.^14,15^ In this study, in addition to artificial alteration, precise control of the quantity of interlayer Al ions was achieved through ion exchange from Na-phlogopite. The ratios of interlayer Al ions (intAl) to the total number of interlayer cations in phlogopite (*r*_intAl_ = intAl/(intAl + K)) were adjusted to 0.95, 0.53, and 0.30 by controlling the salt concentrations during ion-exchange procedures. The interlayer spaces in these samples varied from pillared to partially collapsed. Molecular dynamics (MD) simulations were conducted to analyze Cs^+^ adsorption on partially collapsed HIVs. Molecular simulations of Cs^+^ adsorption on the interlayer wedge regions have been reported in previous research^16^ as a model of interlayered hydroxy-Al; however, the study focused on the interlayer wedge zone without including the hydroxy-Al fragment. To the best of our knowledge, this is the first study that directly simulates Cs^+^ adsorption near hydroxy-Al in interlayers.

## Methods

### Preparation of Artificially Altered Phlogopites

Natural phlogopite (K-Phl), extracted from the Siilinjärvi mine in Finland, was sourced from Repco, Inc. Its chemical compositions are listed in Table 1. Two different methods were employed to prepare the Al-interlayered phlogopite. The first method controls the concentration of interlayer Al^3+^, whereas the second method selectively exchanges weakly adsorbed K^+^ for Al^3+^, a process similar to natural weathering.

**Table 1:** Chemical Compositions of Artificially Altered Phlogopite

Sample	Chemical composition	Layer charge (e^–^/T_4_O_10_)^a^	*r*_intAl_
K-Phl	(K_0.94_Na_0.02_Ca_0.11_)(Mg_2.68_Fe_0.30_Ti_0.01_)(Si_2.98_Al_0.84_ Fe_0.17_)O_10_(OH_1.74_F_0.26_)	1.02	0.00
KAl_27%_-Phl	(K_0.54_Na_0.01_Al_0.20_)(Mg_2.65_Fe_0.33_Ti_0.01_)(Si_2.95_Al_0.92_Fe_0.13_)O_10_(OH_1.76_F_0.24_)	1.05	0.27
KAl_30%_-Phl	(K_0.50_Na_0.01_Ca_0.01_Al_0.21_)(Mg_2.73_Fe_0.25_Ti_0.01_)(Si_3.01_Al_0.78_Fe_0.21_)O_10_(OH_1.74_F_0.26_)	0.99	0.30
KAl_53%_-Phl	(K_0.26_Na_0.01_Al_0.29_)(Mg_2.70_Fe_0.28_Ti_0.01_)(Si_3.00_Al_0.83_Fe_0.17_)O_10_(OH_1.71_F_0.29_)	1.00	0.53
Al-Phl	(K_0.02_Na_0.01_Al_0.37_)(Mg_2.70_Fe_0.28_Ti_0.01_)(Si_3.03_Al_0.78_Fe_0.19_)O_10_(OH_1.74_F_0.26_)	0.97	0.95

a T in T_4_O_10_ denotes the ion at a tetrahedral site.

In the first method, Na-phlogopite (Na-Phl) was prepared by repeatedly exchanging interlayer K^+^ with Na^+^. Specifically, 5 g of K-Phl was stirred in a 2 M NaCl aqueous solution (1 L) at 60 °C for 24 h, followed by filtration. This process was repeated five times using fresh NaCl solutions. Na^+^ was mostly exchanged with Al^3+^ by immersing 4 g of Na-Phl in a 1 M AlCl_3_ aqueous solution (400 mL) for 3 h, and 95% ion-exchanged (*r*_intAl_ = 0.95) Al-phlogopite (Al-Phl) was obtained. Additionally, two partially K^+^ ion-exchanged (*r*_intAl_ = 0.30 and 0.53) Al-phlogopites (KAl_30%_-Phl and KAl_53%_-Phl) were obtained by immersing 4 g of Na-Phl in 3 and 100 mM AlCl_3_ aqueous solutions (400 mL), respectively, for 3 h and substituting the residual Na^+^ with K^+^ in a 100 mM KCl aqueous solution (400 mL). The pH of the suspension for preparing the Al-Phl and KAl_53%_-Phl was 1.64 at 18.4 °C and 3.15 at 19.1 °C, respectively. Therefore, in these suspensions, the precipitation of Al­(OH)_3_ did not occur. The pH of the suspension for preparing KAl_30%_-Phl was 4.77 at 18.3 °C. This pH may cause the partial precipitation of Al­(OH)_3_ before adsorption, but no gibbsite peak was observed in the XRD pattern, and the interlayer distance was expanded compared to K-Phl, indicating that the intercalation was successful in this sample.

In the second method, K-Phl (1 g) was immersed in 80 mL of an acidic (pH = 2) 0.2 M AlCl_3_ solution at 80 °C for 2 days, as reported before.^14^ After equilibration, the solid was separated by filtration and repeatedly washed with water to remove the salts adsorbed on the solid. The second method yielded a partially K^+^ ion-exchanged (*r*_intAl_ = 0.27) Al phlogopite (KAl_27%_-Phl).

The chemical compositions of the phlogopites were measured using inductively coupled plasma optical emission spectrometry (SPS3520UV-DD, Hitachi High-Technologies) by dissolving them in aqueous solutions through acidic and basic treatments. Their chemical compositions are listed in Table 1.

### Cs Adsorption and Desorption Treatments

Samples (0.3 g) of Na-Phl, KAl_30%_-Phl, and KAl_53%_-Phl were each added to 30 mL of a CsCl aqueous solution, and the mixture was shaken for 24 h at room temperature using a horizontal tube rotator. For adsorption experiments, Cs solutions with concentrations of 7.5 × 10^–4^ and 7.5 × 10^–3^ mmol/L were used for the low-concentration range, while a 7.5 mmol/L solution was used to prepare saturated samples for desorption tests. Cs-adsorbed powder samples were obtained by centrifuging the solution, discarding the supernatant, and then drying. The amount of Cs adsorbed was determined by analyzing the concentration of the supernatant, which was filtered through a 0.20-μm syringe filter, by using inductively coupled plasma mass spectrometry (ICPMS-2030, Shimadzu). In the desorption tests, 0.2 g of each powder sample was added to 20 mL of a 3 mol/L Mg­(NO_3_)_2_ aqueous solution and shaken for 24 h at room temperature using a horizontal tube rotator. After shaking, the suspension was centrifuged, and the supernatant was collected for analysis, while the solid was retained. This desorption treatment was repeated twice using fresh Mg­(NO_3_)_2_ aqueous solutions. The concentration of Cs^+^ in the supernatant obtained after each treatment was analyzed by ICP-MS. Finally, the retained solids were dried to obtain powder samples.

### Aging Test of Cs Adsorption

To reveal the aging mechanism of Cs adsorption on hydroxy-Al interlayered phlogopite, 0.3 g of KAl_27%_-Phl was added to 30 mL of a highly concentrated 7.5 mmol/L (1,000 ppm) CsCl aqueous solution and shaken for 24 h at room temperature using a horizontal tube rotator. A dry Cs-adsorbed sample was obtained after these treatments. The sample was then placed in distilled water in a sealed container and heated for 5 days at 180 °C. The sample was dried, and the structural changes in the hydroxy-Al were analyzed using NMR spectroscopy. The details of NMR spectroscopy are explained in the next section.

### Characterization of Artificially Altered Phlogopite

Interlayer distance was measured via the X-ray diffraction (XRD) method using an Ultima IV instrument (Rigaku Corp.), employing Cu Kα radiation at 40 kV and 30 mA. To investigate the effect of water, XRD measurements were performed under low (<2%) and high (75–80%) relative humidity (RH) conditions, as previously reported.^17,18^ To examine the coordination number of oxygen around Al^3+^, solid-state magic angle-spinning nuclear magnetic resonance (MAS NMR) spectra of ^27^Al were measured for altered phlogopite powder in a 3.2 mm sample rotor. The measurements were performed under an applied magnetic field of 18.8 T at room temperature using a single-pulse sequence. Typical pulse widths of 1.1 μs were applied for ^27^Al, corresponding to an approximate π/6 tip-angle pulse relative to a liquid standard. A rotor spinning rate of ∼18 kHz and a repetition time of 2 s were used for ^27^Al.

### Structural Models of HIV

In this simulation, HIV with an *r*_intAl_ = 0.30 was modeled. For simplicity, the layer charge of the model was fixed at 0.7, similar to common vermiculite, and all octahedral Fe^2+^ atoms were substituted by Mg^2+^. The structural models of hydroxy-Al can either be fragments of gibbsite or Al_13_ polymer.^19^ To compare with the interlayer distance of our altered phlogopite (1.39 nm), the gibbsite fragment model was considered the most appropriate. The charge of the gibbsite model depends on the pH of the solution, owing to the adsorption and desorption of protons at the edges. The isoelectric point of the gibbsite edges was reported to be pH = 8–10;^19,20^ therefore, the positively charged Al_24_(OH)_60_^12+^ and neutral Al_24_(OH)_72_ models were considered for acidic (pH < 8–10) and slightly basic (pH = 8–10) conditions, respectively, as shown in Figure 1. The positively charged model corresponds to the previously proposed structure reported in the literature.^19^ The size of the gibbsite fragments may depend on the degree of polymerization, and the shape can be linear or cyclic.^21^ Here, we modeled one of the cyclic polymers. The coordination number of oxygen atoms around the Al^3+^ at the edges can be five (^[5]^Al) or six (^[6]^Al); therefore, four models—Al_24_(OH)_60_^12+^•24H_2_O, Al_24_(OH)_60_^12+^•12H_2_O, Al_24_(OH)_72_•12H_2_O, and Al_24_(OH)_72_—were used in this study.

**Figure 1:**
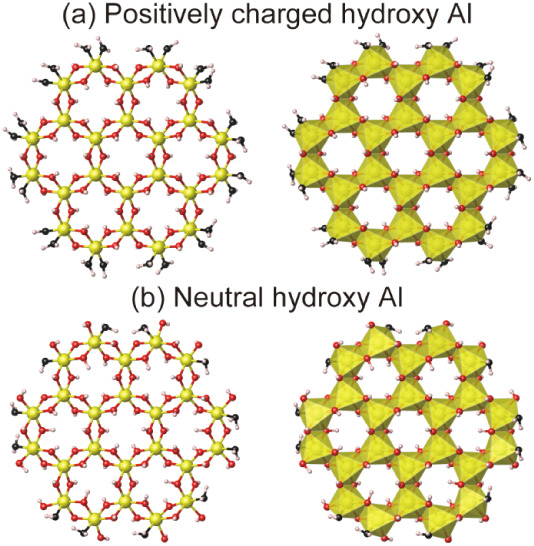
(a) Positively charged hydroxy Al (Al_24_(OH)_60_^12+^•24H_2_O) model for acidic conditions and (b) neutral hydroxy Al (Al_24_(OH)_72_•12H_2_O) model for slightly basic conditions in ball-and-stick view (left) and polyhedral view (right). The coordination number of oxygens around Al ions at the edges is six. White, red, and yellow spheres represent hydrogen, oxygen, and aluminum atoms, respectively. Oxygen of H_2_O molecules are colored black.

The chemical formulas of positively charged and neutral HIV models were determined as follows:

$$\left(K_{0.45}{\mathrm{Cs}}_{0.15}{{\mathrm{Al}}^{\mathrm{hydroxy}}}_{0.10}\right)\left({\mathrm{Mg}}_{3}\right)\left({\mathrm{Al}}_{0.7}{\mathrm{Si}}_{3.3}\right)O_{10}\left({\mathrm{OH}}_{2}\right),\;,\mathrm{at pH}<8-10\;\mathrm{condition}$$

$$\left(K_{0.55}{\mathrm{Cs}}_{0.15}{{\mathrm{Al}}^{\mathrm{hydroxy}}}_{0.10}\right)\left({\mathrm{Mg}}_{3}\right)\left({\mathrm{Al}}_{0.7}{\mathrm{Si}}_{3.3}\right)O_{10}\left({\mathrm{OH}}_{2}\right),\;,\mathrm{at pH}=8-10\;\mathrm{condition}$$

Here, Al^hydroxy^ indicates Al^3+^ in the gibbsite fragments. A structural model of HIV was developed by modifying the structure of donbassite^22,23^ as a reference for the gibbsite sheet embedded between 2:1-type tetrahedral-octahedral-tetrahedral (TOT) silicate layers. All of the octahedral sites in the TOT layers were occupied by Mg^2+^. A negative layer charge is generated by the isomorphic substitution of tetrahedral Si^4+^ with Al^3+^. The supercell contained four TOT layers of HIV. All interlayer cations in the top and bottom interlayers were K^+^. The middle two layers contained Al^hydroxy^, K^+^, and Cs^+^.

### Molecular Dynamics Simulations

The force fields were based on a Born-Mayer-Huggins and exponential functions (BMH-EXP) type model that was successfully used for clay minerals and water.^24–28^ The potential parameters of the HIV models are listed in Table S1. Long-range Coulombic energy was calculated by using the traditional Ewald method. The velocity Verlet algorithm was used to compute the equations of motion with a time increment of 0.4 fs. To avoid the metastable configurations of Cs^+^ and K^+^ in the interlayer space, high-temperature (398.15 K) constant-volume (*NVT*) simulations were conducted while maintaining a wide interlayer space. Subsequently, simulations were performed in an *NPT* ensemble, maintaining a constant number of atoms, temperature (300 K), and pressure (0.1 MPa) using a Gaussian thermostat and scaling barostat. All simulations were performed using an in-house MXDTRICL code.

### Density Functional Theory Calculations

Density functional theory (DFT) calculations were performed using the ORCA 6 program package.^29^ To investigate the Cs^+^ coordination environment and the origin of the five-coordinated aluminum (^[5]^Al) signal, we employed a hydrated gibbsite cluster model (Al_10_O_38_H_46_) consisting of two edge-sharing rings. This model ensures consistency with the structural units observed in our MD simulations. Structural optimizations were carried out at the B97-3c/def2-mTZVP level.^30^ B97-3c is a robust composite method optimized for molecular geometries and noncovalent interactions, which is suitable for characterizing the steric effect of the Cs^+^ cation in Cs-gibbsite models (CsAl_10_O_38_H_45_). The SMD (Water) implicit solvation model^31^ was used to account for the aqueous environment. The ^27^Al NMR chemical shieldings were calculated using the GIAO method^32^ at the ωB97X-D4.rev/def2-TZVPD level.^33^ Range-separated hybrid functionals such as ωB97X-D4 provide high accuracy for spectroscopic properties by reducing self-interaction errors. The inclusion of diffuse functions in the def2-TZVPD basis set is essential for the reliable prediction of ^27^Al chemical shieldings.

## Results and Discussion

### Experimental Section

#### Interlayer Space of Artificially Altered Phlogopite

The interlayer distances *d* of the phlogopites were measured by a Bragg 001 reflection, as shown in Figure 2. The *d* of pure K-phlogopite (K-Phl) was 1.01 nm, consistent with the reported value,^34^ and no swelling was observed even under high RH conditions. By exchanging the interlayer K^+^ with Na^+^ (Na-Phl), the peak broadened at around 1.02 nm under low RH conditions (Figure 2b, left). A sharp peak was observed at a larger interlayer distance of 1.48 nm under high RH conditions (Figure 2b, right), indicating that most K^+^ was replaced by Na^+^ and Na-Phl became expandable by water. By the intercalation of Al^hydroxy^ at *r*_intAl_ = 0.27 (KAl_27%_-Phl, Figure 2c), relatively sharp peaks at 1.37 and 1.01 nm, as well as a broad peak at 1.14 nm, were observed under low RH conditions. A small peak shift was observed from 1.37 and 1.14 nm to 1.39 and 1.16 nm under high RH conditions, while the peak at 1.01 nm remained constant. The interlayer distance of 1.37–1.39 nm reflected the presence of Al^hydroxy^ in the interlayer, consistent with the previously reported value of 1.4 nm.^11^ The peaks at 1.37 and 1.01 nm correspond to the presence of layers bearing only Al^hydroxy^ and K^+^ in the interlayers, respectively. The broad peak at around 1.14 nm is attributed to random interstratification of the Al^hydroxy^ and K^+^-phlogopite layers. The peak shifts under high RH conditions indicate that swelling occurs at the Al^hydroxy^ interlayers. For KAl_30%_-Phl (Figure 2d), a broad peak was observed at 1.09 nm under both low and high RH conditions. This broad peak corresponds to the random interstratification of hydrated Al^3+^-, Al^hydroxy^-, and K^+^-phlogopite layers. The peak was slightly broadened under high RH conditions, indicating that swelling occurs in these layers. For KAl_53%_-Phl (Figure 2e), a broad peak was observed at 1.19 nm with a shoulder at a lower angle. This peak is attributed to hydrated Al^3+^, due to the similar interlayer distance as Al-Phl (Figure 2f), and random interstratification including Al^hydroxy^ and K^+^-phlogopite layers. The peak shifted to 1.39 nm and became sharp under high RH conditions, indicating that swelling occurs in most interlayers.

**Figure 2:**
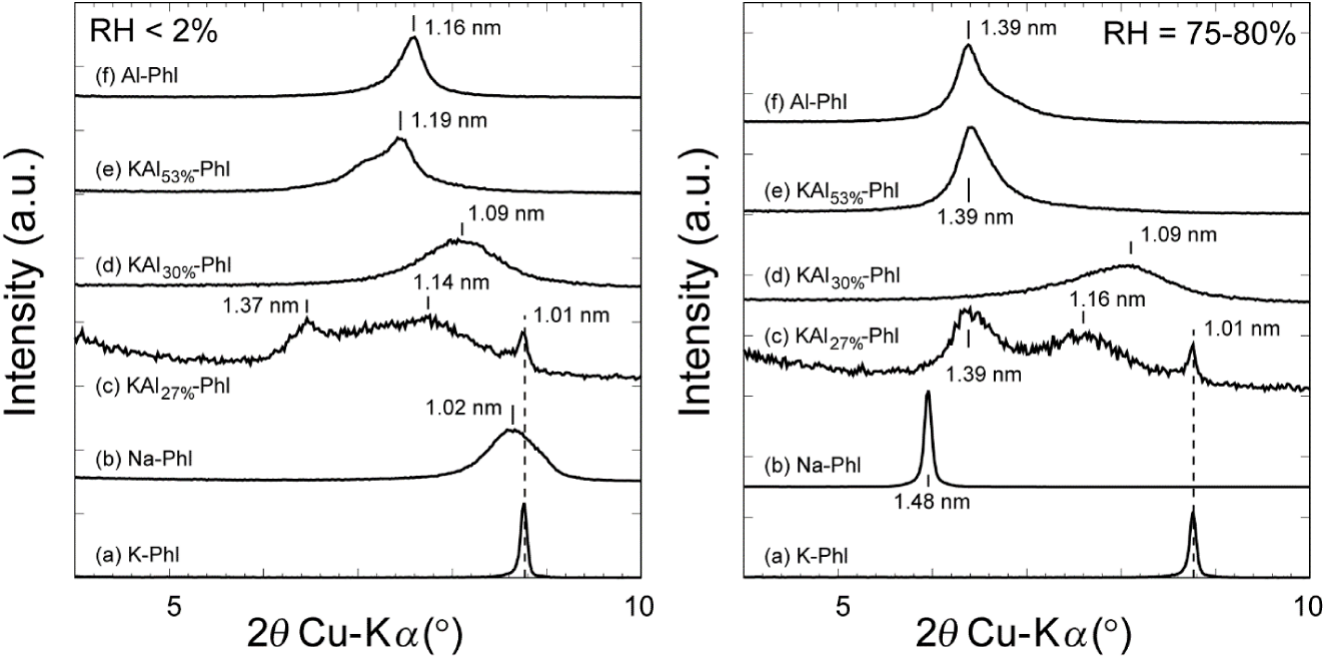
XRD patterns showing the basal reflections of (a) K-Phl, (b) Na-Phl, (c) KAl_27%_-Phl, (d) KAl_30%_-Phl, (e) KAl_53%_-Phl, and (f) Al-Phl at relative humidity (RH) levels of <2% (left) and 75–80% (right). Numbers near the lines indicate the interlayer distances at the corresponding angles.

### Coordination Number around the Interlayer Al Ions in Altered Phlogopites

Al^3+^ can form tetrahedral AlO_4_ or octahedral AlO_6_ configurations. NMR spectra can distinguish this difference.^35,36^ As shown in Figure 3, the ^27^Al MAS NMR spectrum of Na-Phl shows only a single peak at approximately 67 ppm, suggesting that Al^3+^ is present in a tetrahedral configuration, resulting from the isomorphic substitution of Si^4+^ in the phlogopite structure. This also indicates the absence of Al^3+^ in the octahedral sheets of the phlogopites. Conversely, the peak at around 2.5 ppm, which suggests the presence of an octahedral form, was observed for the altered phlogopites (KAl_30%_-Phl and KAl_53%_-Phl), and the intensity increased with increasing interlayered Al^hydroxy^ amount. This indicates that the interlayered Al^3+^ was present in an octahedral form, similar to a fragment of the gibbsite sheet.

**Figure 3:**
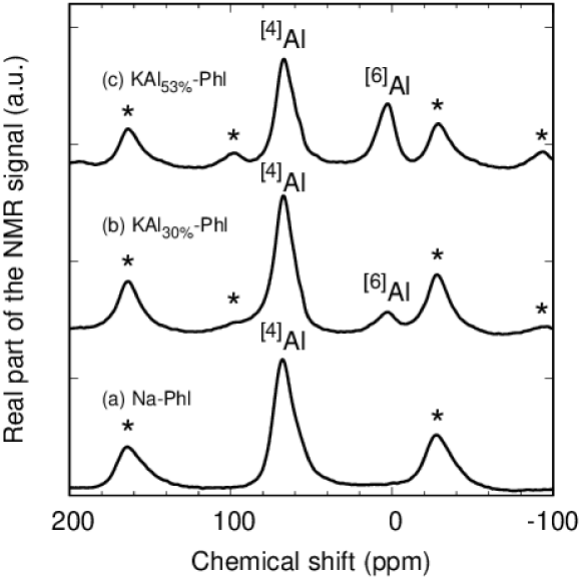
^27^Al magic angle-spinning nuclear magnetic resonance spectra for (a) Na-Phl, (b) KAl_30%_-Phl, and (c) KAl_53%_-Phl. Spinning sidebands are marked by asterisks. ^[4]^Al and ^[6]^Al indicate that the peaks originate from four- and six-coordinated Al^3+^, respectively.

### Structure of Altered Phlogopites

Based on an experimental analysis of the chemical composition, interlayer distance, and chemical species of Al^3+^, schematic figures of the three altered phlogopites are shown in Figure 4. In the unaltered K-Phl, all Al^3+^ was present in tetrahedral sheets of phlogopite, and most of the interlayer cations were K^+^ with an interlayer distance of 1.01 nm. In partially altered KAl_27%_- and KAl_30%_-Phl, the interlayer distance varied from 1.0 to ∼1.39 nm. A large interlayer distance was achieved in the presence of octahedral Al^3+^. By increasing the Al content in the interlayer (KAl_53%_-Phl), an interlayer distance of 1.39 nm was achieved owing to the pillared effect of the octahedral Al^3+^.

**Figure 4:**
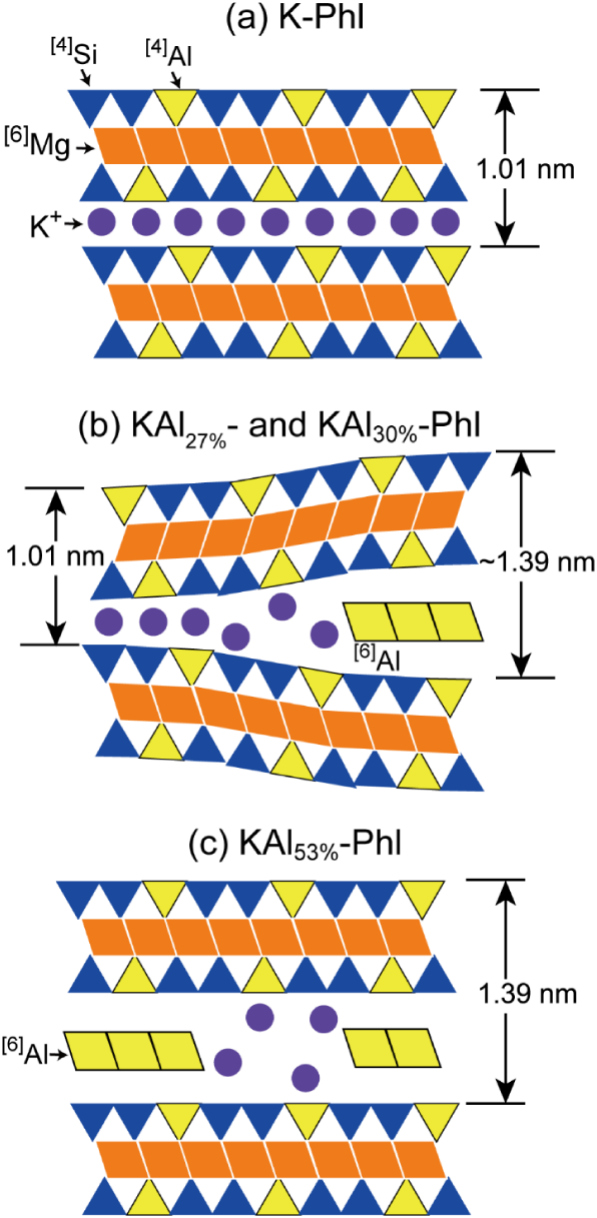
Interlayer structures of (a) K-Phl, (b) KAl_27%_-Phl and KAl_30%_-Phl, and (c) KAl_53%_-Phl estimated from the chemical composition, XRD analysis, and NMR spectra.

### Adsorption and Desorption of Cs^+^

The distribution coefficients (*K*_d_) of Cs^+^ at low concentrations (7.5 × 10^–4^ and 7.5 × 10^–3^ mmol/L) and high concentrations for altered phlogopites in solution are listed in Table 2. The adsorption rates of Cs^+^ on Al^hydroxy^-intercalated phlogopites (KAl_30%_-Phl and KAl_53%_-Phl) were slightly higher than those of K- and Na-Phl at a low Cs concentration of 7.5 × 10^–4^ mmol/L. High *K*_d_ values of the Al^hydroxy^-intercalated phlogopites relative to K- and Na-Phl were observed at higher Cs concentrations. There was no significant difference in the *K*_d_ values of KAl_30%_-Phl and KAl_53%_-Phl.

**Table 2:** Cs^+^ Distribution Coefficients (*K*_d_ (mL/g)) for Four Phlogopite Samples

Solid	*K*_d_ (7.5 × 10^–4^ mmol L^–1^ Cs^+^)	*K*_d_ (7.5 × 10^–3^ mmol L^–1^Cs^+^)	*K*_d_ (7.5 mmol L^–1^ Cs^+^)
K-Phl	9.8 × 10^3^	1.7 × 10^3^	Not measured
Na-Phl	9.1 × 10^3^	1.7 × 10^4^	Not measured
KAl_30%_-Phl	1.3 × 10^4^	9.1 × 10^4^	6.5 × 10^4^
KAl_53%_-Phl	1.3 × 10^4^	9.6 × 10^4^	6.0 × 10^4^

As shown in Figure 5 and Table S2, the Cs desorption rates depended on the number of Al^hydroxy^. The Cs desorption rate of KAl_30%_-Phl was lower than that of KAl_53%_-Phl, indicating that KAl_30%_-Phl strongly fixed Cs^+^.

**Figure 5:**
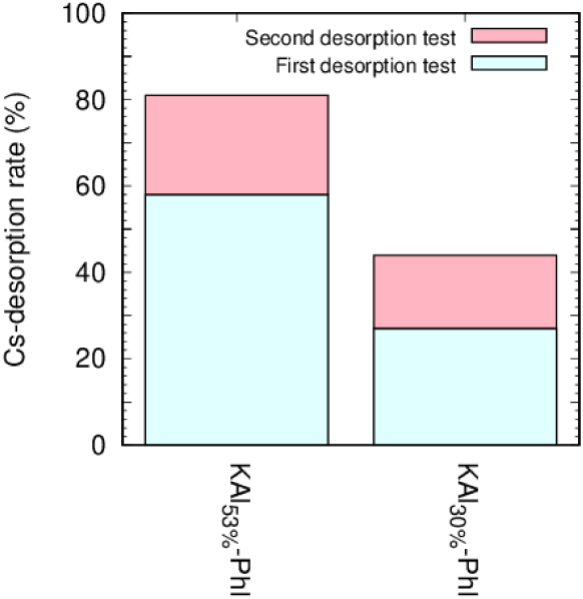
Cs desorption rates from KAl_53%_-Phl (left) and KAl_30%_-Phl (right) after two desorption treatments.

### Change in the Structure of Al^hydroxy^ Due to Cs Adsorption in Aging Test

As shown in Figure 6, a clear change in the ^27^Al NMR spectra of Cs-adsorbed KAl_27%_-Phl was observed. In addition to the peaks of ^[4]^Al and ^[6]^Al, a peak attributable to ^[5]^Al was observed. This peak assignment is consistent with the density functional theory (DFT) evaluation of ^27^Al NMR shielding for the AlO_*x*_ (*x* = 4, 5, and 6) polyhedra, which predicts that ^27^Al chemical shifts of AlO_4_ and AlO_5_ should be ∼ 60 and 25–30 ppm, respectively, relative to those of AlO_6_ (0 ppm) (Figure S1 and Table S3). This implies that the aging effect of Cs may be related to the presence of ^[5]^Al in the interlayer. Another peak observed between ^[5]^Al and ^[6]^Al suggests the presence of distorted AlO_6_ octahedra, caused by the adsorption of Cs^+^.

**Figure 6:**
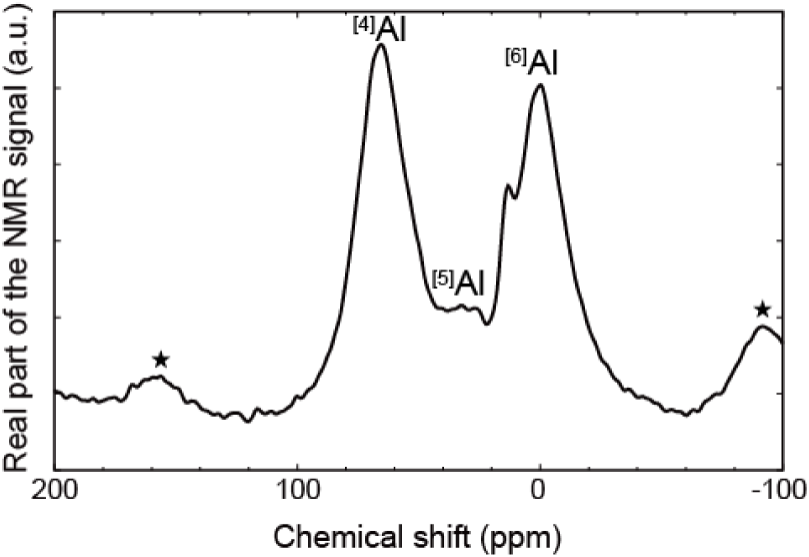
^27^Al magic angle-spinning nuclear magnetic resonance spectrum of KAl_27%_-Phl after Cs adsorption at room temperature, followed by aging at 180 °C for 5 days. Spinning sidebands are marked by asterisks. ^[4]^Al, ^[5]^Al, and ^[6]^Al indicate that the peaks originate from 4-, 5-, and 6-fold coordinated Al^3+^, respectively.

### Structure Model of Interlayer under the Presence of Al^hydroxy^

As shown in Figure 7, the interlayer distances obtained by MD simulations with and without Al^hydroxy^ were 1.4 and 1.1 nm, respectively, consistent with the XRD measurements (Figure 2). Based on these distances, three interlayer zones can be classified as follows: hydroxy-Al interlayered (HI) zone: *z* = 1.4 nm; wedge zone: 1.1 nm < *z* < 1.4 nm; and collapsed zone: *z* = 1.1 nm. This structure supports the hypothesis that the collapse of the interlayer can occur in HIV. The interlayer cations (K^+^ and Cs^+^) were located in the wedge and collapsed zones.

**Figure 7:**
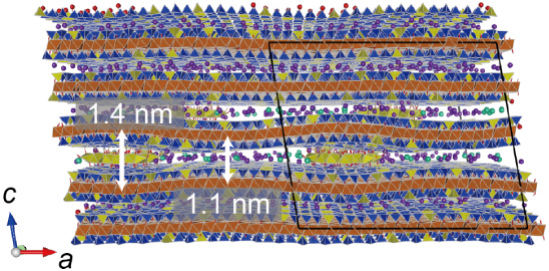
Snapshot of the equilibrium structure of HIV. Solid black lines indicate the boundaries of the supercell. Al, Si, and Mg polyhedra are colored yellow, blue, and brown, respectively. K and Cs ions are colored purple and green, respectively. The equilibrium interlayer distance, including Al^hydroxy^ is 1.4 nm, while that without Al^hydroxy^ is 1.1 nm.

### Adsorption of Cs^+^ in the Neutral Al^hydroxy^ Model

The stabilities of the interlayer cations were qualitatively evaluated based on their trajectories during simulations (Figures 8 and S2). The trajectories of cations in the collapsed layers at interlayer distances, *d*, shorter than 1.1 nm were fixed in the ditrigonal rings of the SiO_4_ sheets, implying that hopping among the ditrigonal rings of cations did not occur during this simulation. The trajectories of cations adsorbed at the edges of the neutral Al^hydroxy^ groups were slightly larger than those in the collapsed layers; however, desorption was not observed in this simulation. On the contrary, the size of the trajectories of cations in the wedge zone at interlayer distances of 1.1–1.2 nm was large, and hopping was observed among the adsorption sites, indicating that these ions were loosely fixed in the site. The mean square displacement (MSD) of K^+^ and Cs^+^ clearly changes depending on the interlayer distances (Figures 8 and S2). In the collapsed layers (*d* < 1.1 nm), most ions were fixed in the ditrigonal rings, resulting in small MSD. In the wedge zone (*d* = 1.1–1.2 nm), the MSD increased for both K^+^ and Cs^+^, although the K^+^ had higher values than the Cs^+^. The small ionic radius of K^+^ caused a decrease in the attractive interaction from either the top or bottom TOT layers, resulting in an increased MSD. Near the edges of Al^hdroxy^ (*d* > 1.2 nm), the MSD at 10 ps is comparable between K^+^ and Cs^+^. This is due to the adsorption of these ions at the edges of Al^hdroxy^.

**Figure 8:**
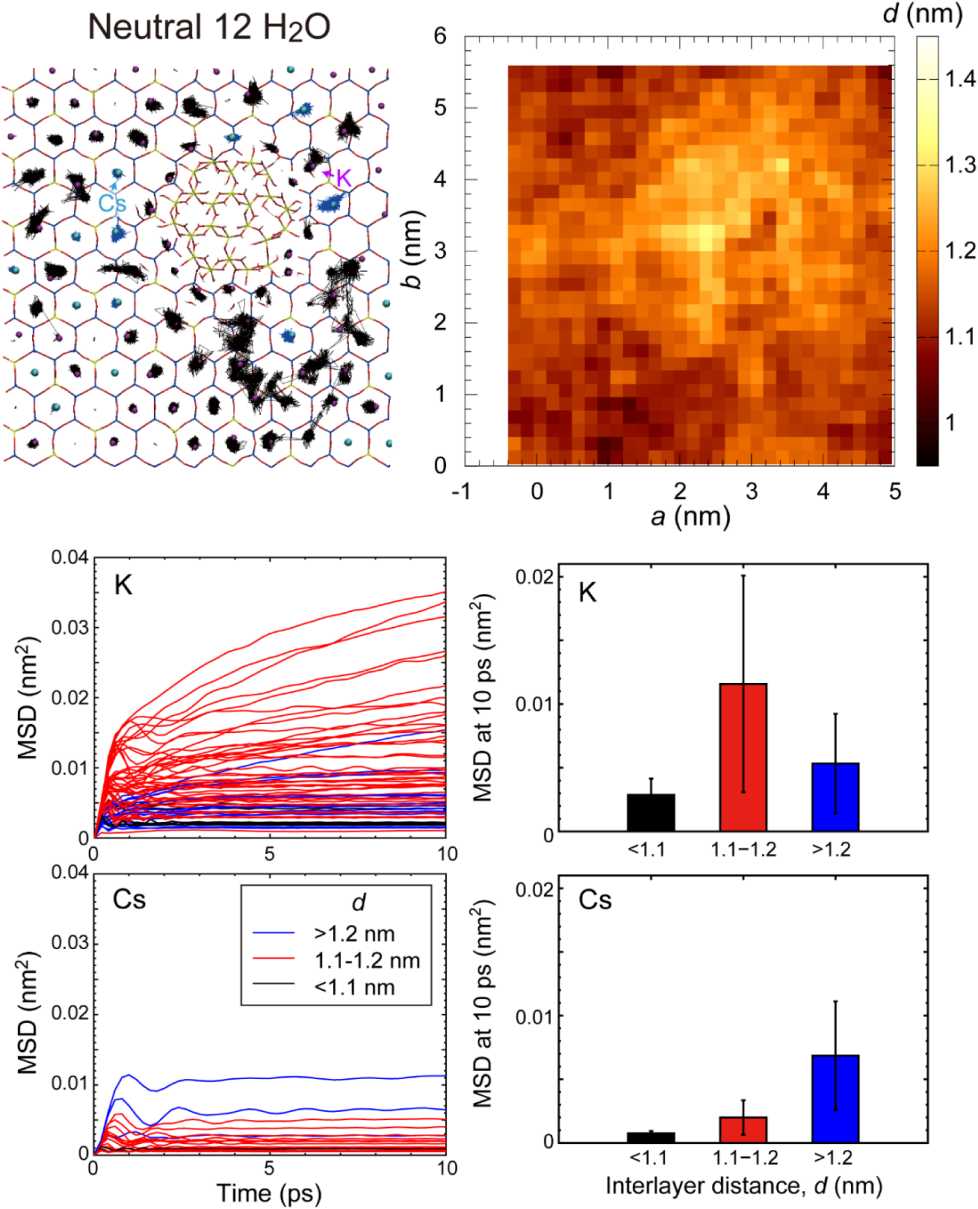
(Top left) Trajectories of interlayer Cs^+^ (blue lines) and K^+^ (black lines) near the neutral hydroxy Al (Al_24_(OH)_72_•12H_2_O) region. (Top right) Their interlayer distances. (Bottom left) The mean square displacement (MSD) of ions. The color indicates the ranges of interlayer distances where ions are positioned. (Bottom right) The average and standard deviations of MSD at 10 ps.

At the edges of Al^hdroxy^, two types of cation adsorption sites were confirmed: a relatively large site coordinated by four oxygen atoms at the edge (site A) and a small site coordinated by three oxygen atoms (site B), as shown in Figure 9. The strong interaction between neutral Al^hydroxy^ and interlayer cations can be explained by electrostatic interactions between the negatively charged TOT layers and interlayer cations. The negative charge of the TOT layers cannot be compensated by the neutral Al^hydroxy^; therefore, the interlayer cations are attracted toward the neutral Al^hydroxy^. Local electrostatic interactions between the edges of Al^hydroxy^ and interlayer cations create two stable sites for the cations at the edges. The MSD of K^+^ and Cs^+^ near the edges (*d* > 1.2 nm) of the five-oxygen-coordinated Al^hydroxy^ (^[5]^Al^hydroxy^; Figure S2) are slightly smaller than those near the edges of the six-oxygen-coordinated Al^hydroxy^ (^[6]^Al^hydroxy^; Figure 8), indicating that cations near ^[5]^Al^hydroxy^ are slightly more stable than those near ^[6]^Al^hydroxy^.

**Figure 9:**
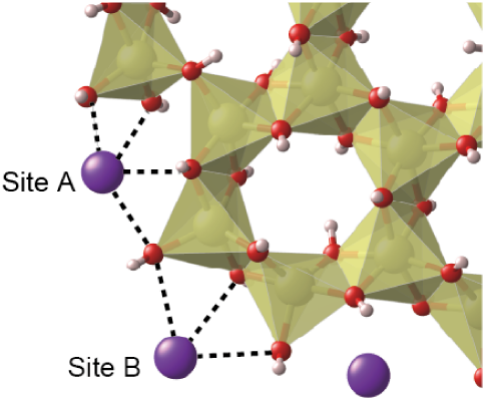
Two stable sites for cations at the edge of neutral Al^hydroxy^. Dotted lines are guides for the eyes to understand the coordination of the nearest oxygen atoms.

### Adsorption of Cs^+^ in the Positively Charged Al^hydroxy^ Model

The adsorption behavior of cations at the edges of positively charged Al^hydroxy^ groups (Figures 10 and S3) was clearly different from that at neutral Al^hydroxy^ (Figures 8 and S2). No Cs^+^ or K^+^ ions were found at adsorption sites near the edge of Al^hydroxy^. This can be explained by the lack of electrostatic attraction between the interlayer cations and negatively charged tetrahedral sheets, because the negative charge is compensated for by the positive Al^hydroxy^. The cation trajectory was large in the wedge zone, as observed in the neutral Al^hydroxy^ model. The MSD at 10 ps increased with increasing interlayer distance for both K^+^ and Cs^+^. The number of ions near the edge of Al^hydroxy^ at large interlayer distances (*d* > 1.2 nm) was small, and these ions were highly mobile.

**Figure 10:**
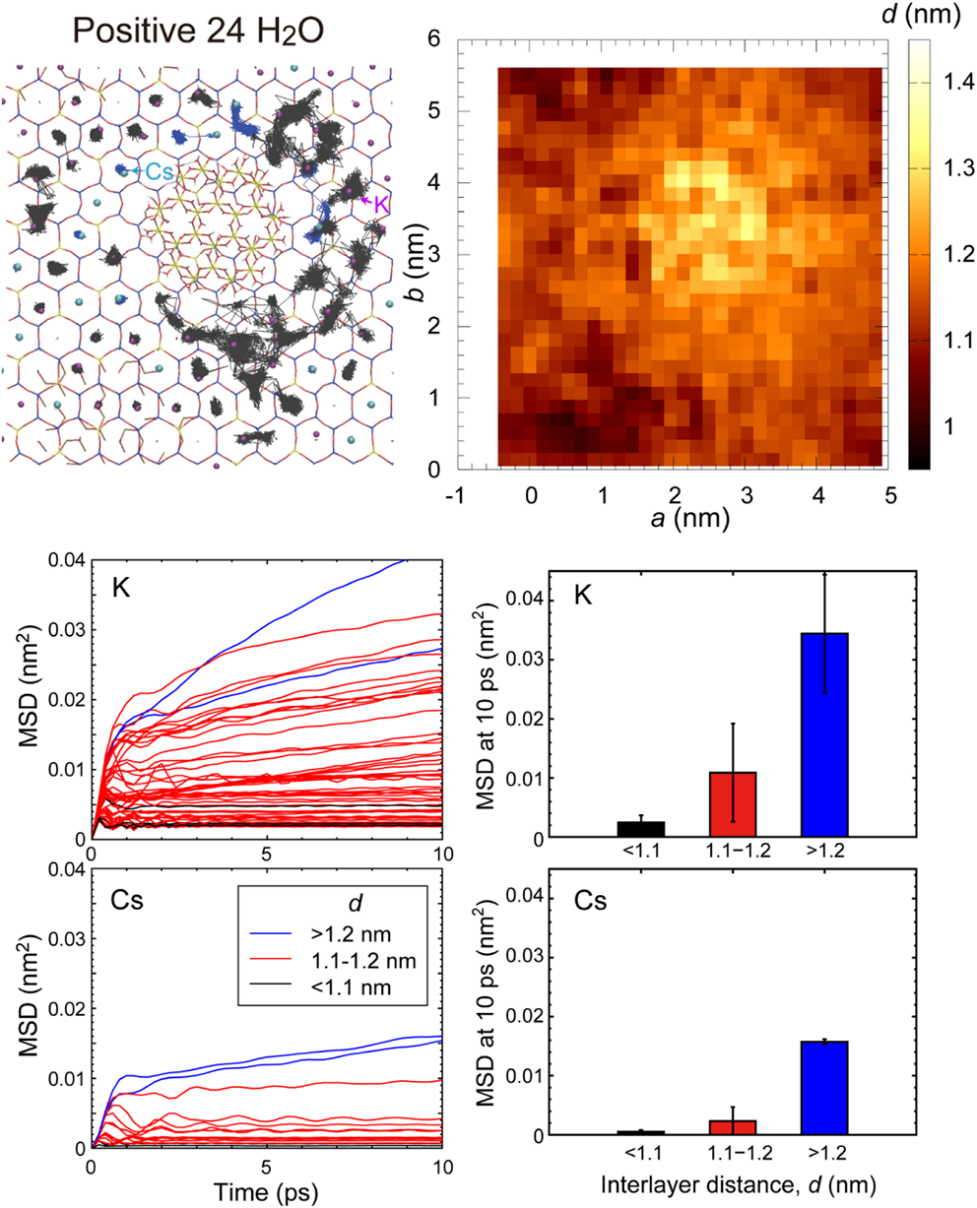
(Top left) Trajectories of interlayer Cs^+^ (blue lines) and K^+^ (black lines) near the positively charged hydroxy Al (Al_24_(OH)_60_^12+^•24H_2_O) region. (Top right) Their interlayer distances. (Bottom left) The mean square displacement (MSD) of ions. The color indicates the ranges of interlayer distances where ions are positioned. (Bottom right) The average and standard deviations of MSD at 10 ps.

### Adsorption of Cs^+^ on Inner Al^hydroxy^

The adsorption of Cs^+^ on the edges of neutral Al^hydroxy^ was found to be stable; however, the difference in fixation between ^[5]^Al^hydroxy^ and ^[6]^Al^hydroxy^ at the edges appeared insufficient to explain the aging effect of Cs adsorption associated with the presence of ^[5]^Al^hydroxy^. Hence, we simulated the trajectory of Cs^+^ within the vacant space of Al^hydroxy^, as shown in Figure 11. Owing to the large ionic radius of Cs^+^, one Al–O bond was broken, resulting in a change in the Al coordination from six to five. If this structure is stable, then the model explains both the presence of ^[5]^Al in the Cs-adsorbed sample and the aging behavior of Cs^+^ within the stable vacant space of Al^hydroxy^.

**Figure 11:**
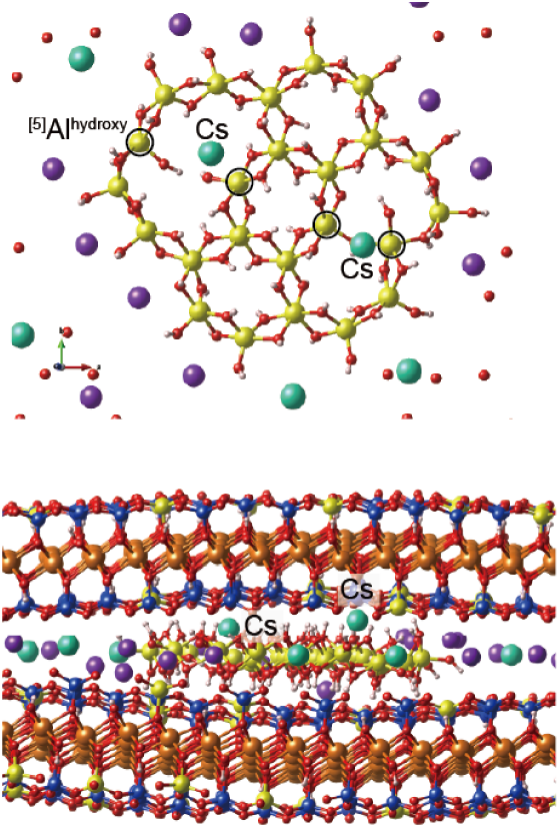
The stable structure of Cs in Al-hydroxy shown from the top view (top) and side view (bottom). ^[5]^Al^hydroxy^ sites formed due to the presence of Cs^+^ are indicated by solid circles.

### Stability of a Cs^+^ on Inner Al^hydroxy^

To elucidate the structural origin of the ^[5]^Al signal observed in the NMR spectrum after the aging process (Figure 6) and a model from MD simulations (Figure 11), we performed DFT calculations to investigate the adsorption of Cs^+^ on the interlayer Al-hydroxy structure. Three representative adsorption configurations were modeled: Cs^+^ located at the edge of the Al-hydroxy hexamer (“Edge” model), above the center of the gibbsite-like ring (“On-top” model), and immersed into the ring (“Inside” model) (Figure 12). An alternative model involving dehydration was also considered; however, the estimated dehydration energies suggest that this process is unlikely to occur spontaneously under the experimental conditions of 180 °C (see the Supporting Information, Table S4, and Figure S4).

**Figure 12:**
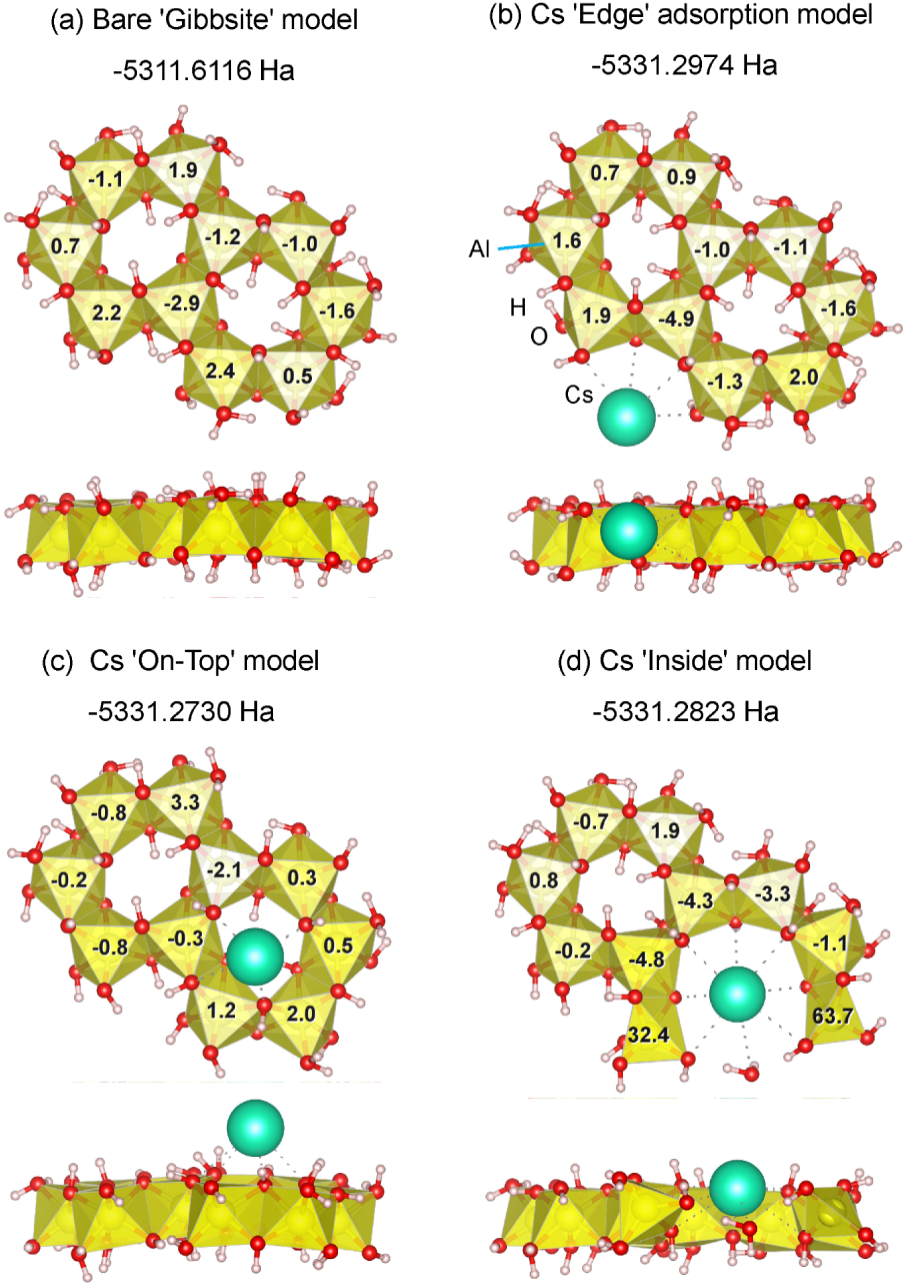
Optimized structures and calculated ^27^Al NMR chemical shifts for hydrated gibbsite clusters (Al_10_O_38_H_46_ and CsAl_10_O_38_H_45_): (a) bare “Gibbsite” model, (b) “*Edge*” adsorption, (c)’*On-Top*’ adsorption, and (d) “*Inside*” fixation site where the 6AlO_6_-ring is cleaved. Numbers indicate NMR chemical shifts relative to the bare model average (557.6 ppm) for ^27^Al. In (d), the large Cs^+^ cation acts as a steric “*wedge*” or a nucleus for local AlO*_n_* reorganization/growth during aging, yielding AlO_5_-like (∼33 ppm) and AlO_4_-like (∼64 ppm) environments.

Geometry optimization of the models indicates that the “Edge” configuration is the most stable, with a total energy of −5331.2974 Ha, suggesting that the “Edge” site is the primary thermodynamic sink for Cs^+^ adsorption. Regarding the “On-top” model, although the protruding Cs^+^ does not seem well stabilized within this cluster model, it might be partially stabilized in an actual interlayer environment by the influence of hollow sites in the adjacent tetrahedral sheets of phlogopite. However, this configuration does not induce any structural rearrangements that could account for the ^[5]^Al signal observed in the NMR spectra. In contrast, the “Inside” configuration was identified as a metastable state with a total energy of −5331.2823 Ha, which is 39.7 kJ/mol (0.0151 Ha) higher in energy than the “Edge” configuration.

This significant energy difference, which is substantially larger than the thermal energy at room temperature (*k*_B_ *T* ∼ 2.5 kJ/mol at 25 °C), indicates that the population of Cs^+^ at the “Inside” site should be negligible under thermodynamic equilibrium. However, the migration from the “Edge” to the “Inside” site could become kinetically accessible under certain conditions. The activation energy (*E*_a_) for this migration must be at least as large as the energy difference of 39.7 kJ/mol. By assuming this value as a conservative lower bound for *E*_a_, the transition rate, estimated using the Arrhenius factor ∼ exp­(−*E*_a_/*k*_B_ *T*), would be more than 2 orders of magnitude greater at 180 °C (*k*_B_ *T* ∼3.8 kJ/mol) than at room temperature. This estimation implies that the migration of Cs^+^ into the “Inside” site, while kinetically hindered at room temperature, could be facilitated by conditions such as hydrothermal treatment or long-term aging.

It should be noted that the structural optimization of the “Inside” model reveals a significant local rearrangement of the Al-hydroxy structure (Figure 12d). The large ionic radius of the Cs^+^ (1.67Å/1.74Å/1.88 Å for coordination number VI/VIII/XII)^37^ exerts a strong steric effect—a “wedge effect”—within the confined ring structure. This effect should induce the cleavage of an edge-sharing Al–O–Al linkage, leading to the formation of five-coordinated AlO_5_ and/or four-coordinated AlO_4_ sites, as shown in Figure 12d. While this structural change can be interpreted as a partial cleavage of the Al-hydroxy ring due to Cs^+^ insertion, it may also represent an early stage of the formation of a gibbsite-like sheet, where the Cs^+^ attached to the edge acts as a nucleus for further growth. The calculated NMR chemical shift for this newly formed AlO_5_ site is approximately 33 ppm, which is in good agreement with the experimental ^[5]^Al peak observed after aging.

### Implications for the Adsorption and Desorption of Cs^+^ in HIV

In the adsorption experiments, the adsorption rates of the Al^hydroxy^-intercalated phlogopites (KAl_30%_-Phl and KAl_53%_-Phl) were higher than those of collapsed K-Phl (Table 2), implying that most of the adsorption sites were present in the interlayer space. The interlayer space available for large-ionic-radius Cs^+^ is essential for adsorption, and a large interlayer distance was observed for KAl_30%_-Phl and KAl_53%_-Phl. The Cs desorption rate of KAl_30%_-Phl was clearly lower than that of KAl_53%_-Phl, indicating two possibilities: the presence of strong attractive forces between KAl_30%_-Phl and Cs ions and/or the presence of specific adsorption sites in KAl_30%_-Phl.

MD simulations of the Al^hydroxy^ groups in the interlayer revealed the presence of strong adsorption sites at the edge of neutral Al^hydroxy^. However, this strong attractive force cannot explain the difference between KAl_30%_-Phl and KAl_53%_-Phl, because adsorption sites can be present in both altered phlogopites. The major difference between these altered phlogopites was their interlayer structures, as shown in Figure 4. The wedge zone, with its slightly open interlayer spacing, might be optimal for the adsorption of large Cs^+^, analogous to the FES.^9^ However, the MD trajectory of ions in the wedge sites indicates that these ions are nonadsorbing and mobile. Therefore, the wedge zone is not a strong adsorption site for the Cs^+^.

We suppose that the partial collapse occurring in the wedge zone can contribute to the low desorption rate of KAl_30%_-Phl and may be a mechanism for the aging of Cs adsorption in natural clay minerals. The presence of a wedge zone also helps Cs^+^ penetrate deeply into the interlayer space by enhancing its mobility. Furthermore, the vacant space within Al^hydroxy^ is another potentially stable site. These sites can be stable, as implied by DFT calculations, and they may account for the presence of ^[5]^Al in conjunction with the aging of Cs adsorption.

The adsorption mechanism of Cs^+^ examined in this study is summarized in Figure 13. First, Cs^+^ are adsorbed in the interlayer space, and some Cs^+^ are strongly adsorbed at the edges of the neutral Al^hydroxy^. In the wedge zone, cations are loosely adsorbed and can exchange with other cations. Over time, some Cs^+^ may occasionally migrate into the collapsed zone (fixation model 1) or into Al^hydroxy^ (fixation model 2), where they become strongly fixed.

**Figure 13:**
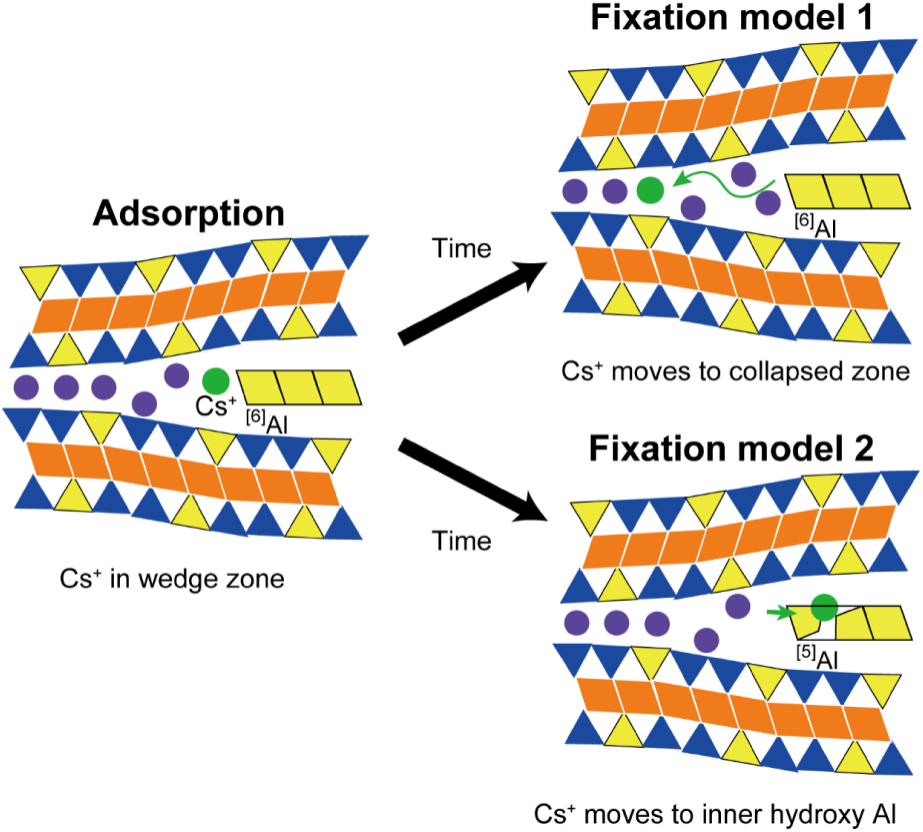
A schematic of two plausible Cs adsorption and fixation (aging) processes in HIV.

## Conclusions

The adsorption and desorption behaviors of Cs^+^ in hydroxy-Al-interlayered vermiculite were studied to elucidate the aging effect. The high Cs^+^ distribution coefficients (*K*_d_) of the hydroxy-Al interlayered phlogopites at low Cs^+^ concentrations (<7.5 × 10^–3^ mmol L^–1^) indicate high selectivity compared to Na- and K-phlogopites. The low Cs^+^ desorption rates from low-concentration hydroxy-Al phlogopite suggest the presence of strong adsorption sites. In the aging test of Cs adsorption, the 5-fold coordination of Al was observed in the NMR spectra. Molecular dynamics simulations revealed two strong adsorption sites in the hydroxy-Al-interlayered phlogopite: one near the edge of the electrically neutral hydroxy-Al sheet and one within the collapsed interlayer. Unexpectedly, the Cs^+^ in the wedge zone between the hydroxy-Al and collapsed zones were found to be highly mobile, indicating that this zone cannot effectively fix the Cs^+^. To explain the experimentally observed effects of aging on Cs^+^ fixation, we proposed a two-step mechanism. First, Cs^+^ penetrates the vermiculite interlayer because of the large interlayer space, expanded by the presence of hydroxy-Al segments. Weak attractive interactions in the wedge zone facilitate this penetration. Second, the Cs^+^ gradually migrate into the collapsed zone via ion exchange or become trapped within the inner vacant space of Al^hydroxy^. Once the Cs^+^ atoms are fixed at these sites, desorption becomes difficult.

## Supplementary Material



## Notes

Potential functions and parameters used in molecular dynamics simulations, computational methods of NMR chemical shift simulations using DFT, stability of a dehydrated gibbsite model for Cs^+^ adsorption using DFT calculations, and MSD of interlayer ions near two gibbsite models (PDF)
