# Supplementary material for: Adsorption of Cs Ions in Hydroxy-Al Interlayered Clay Minerals and the Aging Mechanism
Source: Langmuir. 2026 Feb 21;42(8):6081–90. doi: 10.1021/acs.langmuir.5c05291 (PMC12961925; doi:10.1021/acs.langmuir.5c05291)
Supplement: Supplementary file 1 [file la5c05291_si_001.pdf]

Supporting Information

Adsorption of Cs Ions in Hydroxy-Al Interlayered Clay Minerals and the Aging Mechanism

Hiroshi Sakuma,[*,§] *Kenji Tamura,*[*,§] *Shigeru Suehara,*[§] *and Kenjiro Hashi*[¶]

[§]National Institute for Materials Science, 1-1 Namiki, Tsukuba, Ibaraki 305-0044 JAPAN

[¶]National Institute for Materials Science, 3-13 Sakura, Tsukuba, Ibaraki 305-0003 JAPAN

KEYWORDS Frayed edge, wedge zone, Cs fixation, aging effect, clay minerals

List of Contents

Potential functions and parameters used in molecular dynamics simulations

Table S1. Potential parameters used in molecular dynamics simulations

Adsorption and desorption of Cs^+ on hydroxy-Al intercalated phlogopites

Table S2. Measured adsorbed and desorbed Cs^+ on hydroxy-Al intercalated phlogopites.

AlO_x (*x*=4,5, and 6) NMR chemical shift simulation by the density functional theory

Figure S1. Muscovite-based models for AlO_x (*x*=4, 5, and 6) NMR simulation. AlO_5 structural units appear in the muscovite AlO_6 octahedral layer after the removal of a hydroxyl group (b), and further removal of a hydrogen atom leads to structure (c).

Table S3. Theoretical ^{27}Al NMR chemical shifts (δ_{iso}) for AlO_x polyhedra (ppm)

Trajectories and MSD of interlayer ions as a function of interlayer distance

Figure S2. (Top left) Trajectories of interlayer Cs (blue lines) and K ions (black lines) near the neutral hydroxy Al ($Al_{24}(OH)_{72} \bullet 0H_2O$) region. (Top right) Their interlayer distances. (Bottom left) Mean square displacement (MSD) of ions. The color indicates the ranges of interlayer distances where ions are positioned. (Bottom right) The average and standard deviations of MSD at 10 ps.

Figure S3. (Top left) Trajectories of interlayer Cs (blue lines) and K ions (black lines) near the Positively charged hydroxy Al ($Al_{24}(OH)_{60}^{12+} \bullet 12H_2O$) region. (Top right) Their interlayer distances. (Bottom left) Mean square displacement (MSD) of ions. The color indicates the ranges of interlayer distances where ions are positioned. (Bottom right) The average and standard deviations of MSD at 10 ps.

DFT Calculations on Dehydrated Models

Table S4. Calculated total energies and dehydration energies (ΔE_{dehyd}) for hydrated (original Cs^+–Gibbsite) and dehydrated models.

Figure S4. Optimized structures and calculated ^{27}Al NMR chemical shifts for dehydrated gibbsite clusters with Cs^+ ($CsAl_{10}O_{37}H_{43}$): (a) '*Edge*' adsorption, (b) '*On-Top*' adsorption, and (c) '*Inside*' fixation site models. To investigate structural dehydration, these models were obtained by removing a water molecule formed from two structural hydroxyl groups within the $6AlO_6$ ring from the presented configurations shown in Figure 12, while

keeping the neutralizing water molecules at the edges intact. Numbers indicate NMR chemical shifts relative to the bare model (Figure 12(a)) average (557.6 ppm) for ^{27}Al. Note that while these dehydrated configurations seem to reproduce the experimental $^{[5]}$Al NMR signals (20-35 ppm), all these dehydrated configurations are energetically unfavorable compared to the hydrated states (see Table S4). Level of theory: ωB97X-D4.rev/def2-TZVPD with SMD (Water).

Potential functions and parameters used in molecular dynamics simulations

The potential functions employed here were the same as those previously reported.[1–7] Briefly, the interatomic potential energy of two-body terms between *i* and *j* atoms, U_{ij}, is expressed as the following equation:

$$U_{ij}\left(r_{ij}\right) = \frac{1}{4\pi\epsilon_0}\frac{z_i z_j e^2}{r_{ij}} + f_0\left(b_i + b_j\right)\exp\left(\frac{a_i + a_j - r_{ij}}{b_i + b_j}\right) - \frac{c_i c_j}{r_{ij}^6} + D_{1ij}\exp\left(-\beta_{1ij}r_{ij}\right) + D_{2ij}\exp\left(-\beta_{2ij}r_{ij}\right) + D_{3ij}\exp\left[-\beta_{3ij}\left(r_{ij} - r_{3ij}\right)^2\right] \quad (1)$$

Here, the first term is the Coulomb interaction, the second is the short-range repulsion, the third is the van der Waals interaction, and the others are related to the radial covalent bond. In these terms, ε_0 is the permittivity of a vacuum, z_i the partial charge of atom *i*, *e* the elementary charge, r_{ij} the distance between atom *i* and *j*, f_0 the constant (= 41.865 kJ nm^{-1}mol^{-1}), a_i the repulsion diameter, b_i the softness coefficient, and c_i the van der Waals coefficient of atom *i*. The parameters for adjusting the radial covalent bonds are D_{1ij}, β_{1ij}, D_{2ij}, β_{2ij}, D_{3ij}, β_{3ij}, and r_{3ij}.

The potential energy of the three-body term U_{ijk} is expressed as follows:

$$U_{ijk}\left(\theta_{ijk}, r_{ij}, r_{jk}\right) = -f\left\{\cos\left[2\left(\theta_{ijk} - \theta_0\right)\right] - 1\right\}(k_1 k_2)^{1/2} \quad (2)$$

The parameter θ_{ijk} denotes the angle among the atoms *i*–*j*–*k*; θ_0, g_r, and r_m are the parameters for adjusting the angular part of covalent bonds.

The potential parameters were listed in Table S1.

Table S1. Potential parameters used in molecular dynamics simulations

Atom	Label	*z* [*e*]	Atomic weight [g/mol]	*a* [nm]	*b* [nm]	*c* [(kJ/mol)$^{1/2}$(nm)^3]
Phlogopite						
O	O	-1.20625	16.00	0.1868	0.0151	0.0560600

Mg	Mg	1.52000	24.305	0.1137	0.0101	0.0040920
Si	Si	2.10000	28.09	0.0987	0.0083	0.0000000
Al	Al	1.95000	26.98	0.1089	0.0088	0.0000000
H	H	0.46000	1.01	0.0074	0.0032	0.0000000
K	K	1.00000	39.1	0.1546	0.0115	0.0286438
Hydroxy-Al						
Al	$Al_{hydroxy}$	1.95000	26.98	0.1089	0.0088	0.0000000
O	$O_{hydroxy}$	-1.04000	16.00	0.1868	0.0151	0.0560600
H	$H_{hydroxy}$	0.46000	1.01	0.0074	0.0032	0.0000000
Water						
O	O_{H2O}	-0.92	16.00	0.17280	0.01275	0.05606
H	H_{H2O}	0.46	1.01	0.00350	0.00440	0.00000

Two body terms for covalent bond

Atom pairs	D_{1ij} [kJ/mol]	β_{1ij} [nm^{-1}]	D_{2ij} [kJ/mol]	β_{2ij} [nm^{-1}]	D_{3ij} [kJ/mol]	β_{3ij} [nm^{-2}]	r_{3ij} [nm]
O-Si	205953.66	50.00	-13734.43	22.40	-	-	-
O-Al	151535.01	50.00	-8104.19	22.40	-	-	-
O-H	57394.93	74.00	-3277.68	31.30	34.74	1280.00	0.1283
$O_{hydroxy}$-$Al_{hydroxy}$	151535.01	50.00	-8104.19	22.40	34.74	2000.00	0.2300
$O_{hydroxy}$-$H_{hydroxy}$	57394.93	74.00	-3277.68	31.30	34.74	1280.00	0.1283
O_{H2O}-$Al_{hydroxy}$	151535.01	50.00	-8104.19	22.40	34.74	2000.00	0.2300
O_{H2O}-H_{H2O}	57394.93	74.00	-2189.30	31.30	34.74	1280.0	0.1283

Three body terms for covalent bond

Atom pairs	f [10^{-22} kJ]	θ [deg.]	r_m [nm]	g_r [nm^{-1}]	

Si-O-Si	0.610	120.00	0.1770	168.0	
H_{H2O}-O_{H2O}-H_{H2O}	1.15	99.50	0.1430	92.0	

Adsorption and desorption of Cs^+ on hydroxy-Al intercalated phlogopites

Table S2. Measured adsorbed and desorbed Cs^+ on hydroxy-Al intercalated phlogopites.

Adsorbent	Initial Cs^+ (ppm)	Adsorbed Cs^+ (μmol/g)	Adsorption rate (%)	First desorbed Cs^+ (μmol/g)	Second desorbed Cs^+ (μmol/g)
$KAl_{30\%}$-Phl	0.8431	0.6334	99.85	0.1710	0.1077
$KAl_{53\%}$-Phl	0.8431	0.6333	99.83	0.3673	0.1457

AlO_x (x=4,5, and 6) NMR chemical shift simulation by the density functional theory

Theoretical predictions of the ^{27}Al NMR chemical shifts were carried out using the GIPAW (Gauge Including Projector Augmented Wave) approach, as implemented in the VASP package.[8–10] The AlO_x (x=4, 5, and 6) polyhedron models were constructed using the $2M_1$ Muscovite structure ($K_4Al_8(OH)_8[Al_4Si_{12}O_{40}]$) as shown in Figure S1(a). The AlO_5 structure was obtained by removing an OH group from an AlO_6 octahedron, resulting in adjacent AlO_5 polyhedra. Then, two AlO_5 configurations were investigated: (1) The Muscovite OH-removed model, where the shared oxygen atom retains its associated hydrogen atom (Figure S1(b)), and (2) the Muscovite H- & OH-removed model, where the hydrogen also removed to recover a two-coordinated, i. e., corner-sharing Al-O-Al bridge (Figure S1(c)). The intrinsic AlO_4 and AlO_6 local served as reference polyhedra in each model. For full structural optimization of these models under periodic boundary conditions, a cutoff energy of 400 eV and a 4×2×1 k-point sampling were employed. For the GIPAW NMR shielding evaluations, a higher cutoff energy of 600 eV and a denser 8×4×2 k-point mesh were applied. The isotropic chemical shift (δ_{iso}) is shown in Table S3.

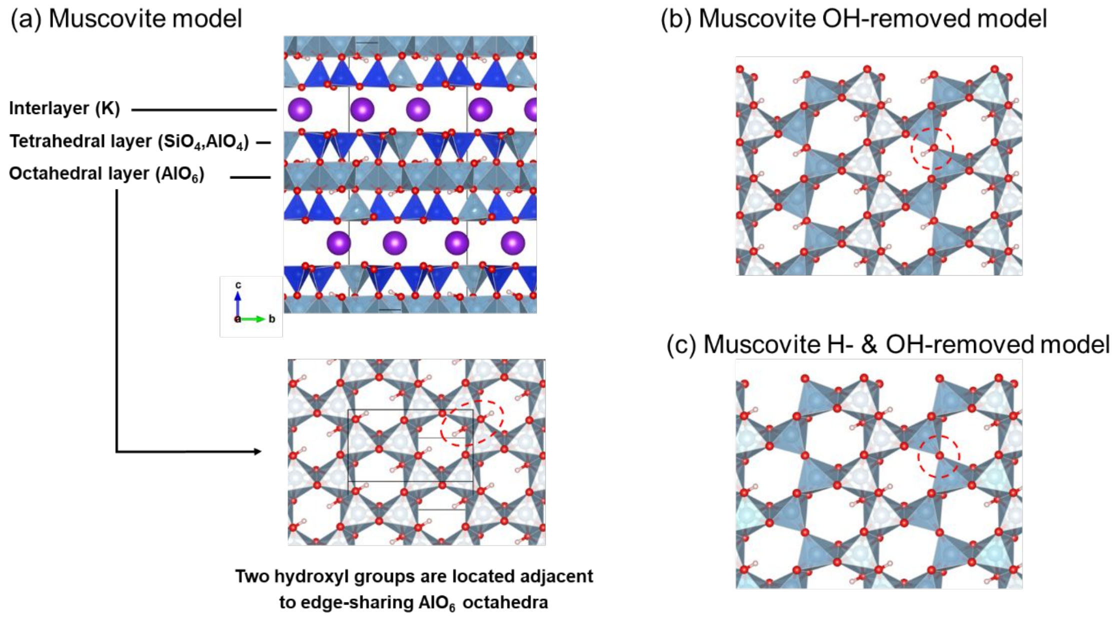

Figure S1. Muscovite-based models for AlO_x (x=4, 5, and 6) NMR simulation. AlO_5 structural units appear in the muscovite AlO_6 octahedral layer after the removal of a hydroxyl group (b), and further removal of a hydrogen atom leads to structure (c).

Table S3. Theoretical ^{27}Al NMR chemical shifts (δ_{iso}) for AlO_x polyhedra (ppm)

Model	AlO_4	AlO_5	AlO_6
Muscovite (No AlO_5)	61.04	N/A	0 (std)
Muscovite OH-removed model	59.80	28.56	0 (std)
Muscovite H- & OH-removed model	60.67	26.44	0 (std)
Suggested range for δ_{iso}	~ 60	25--30	0 (std)

Trajectories and MSD of interlayer ions as a function of interlayer distance

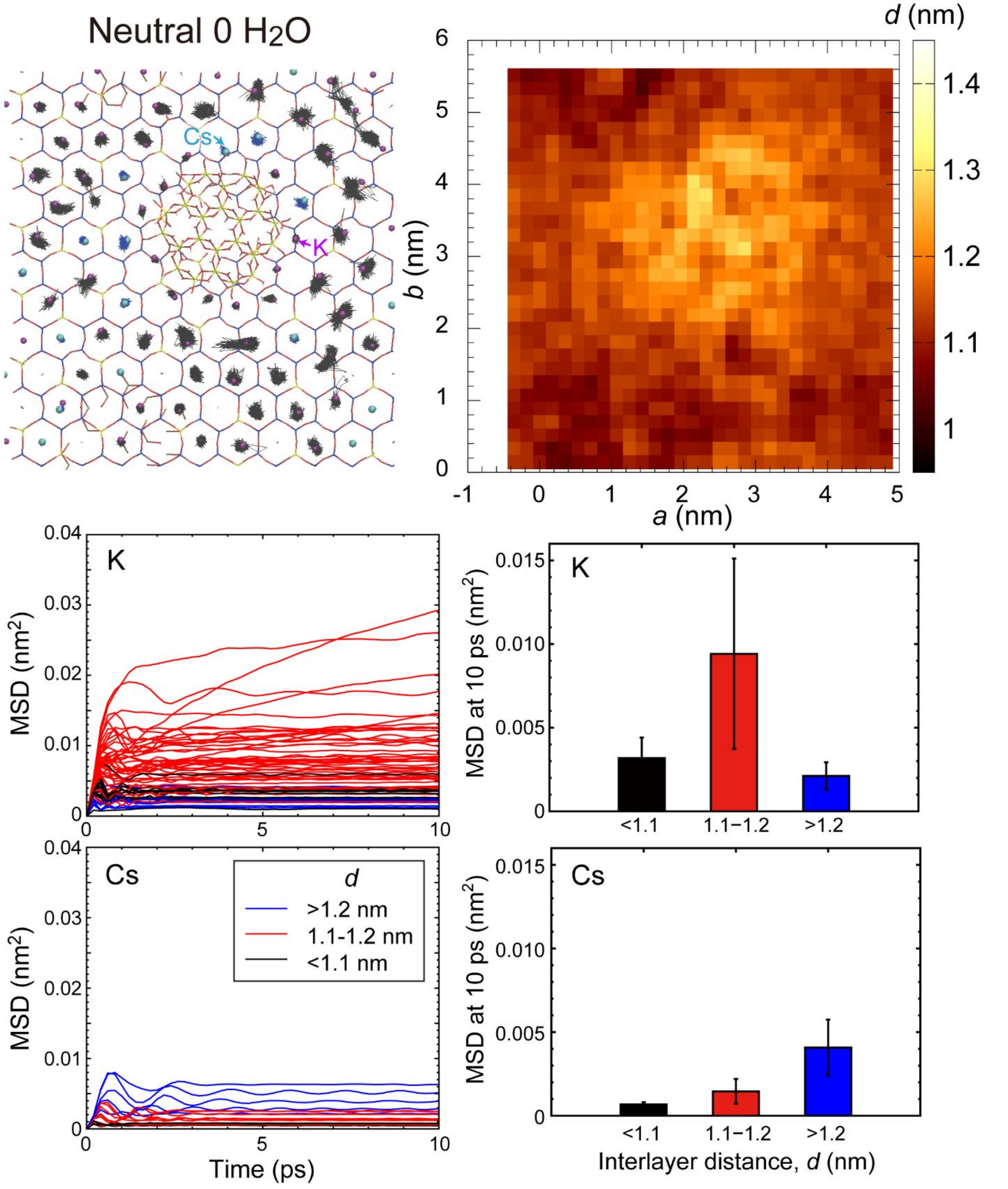

Figure S2. (Top left) Trajectories of interlayer Cs (blue lines) and K ions (black lines) near the neutral hydroxy Al ($Al_{24}(OH)_{72} \cdot 0H_2O$) region. (Top right) Their interlayer distances. (Bottom left) Mean square displacement (MSD) of ions. The color indicates the ranges of interlayer distances where ions are positioned. (Bottom right) The average and standard deviations of MSD at 10 ps.

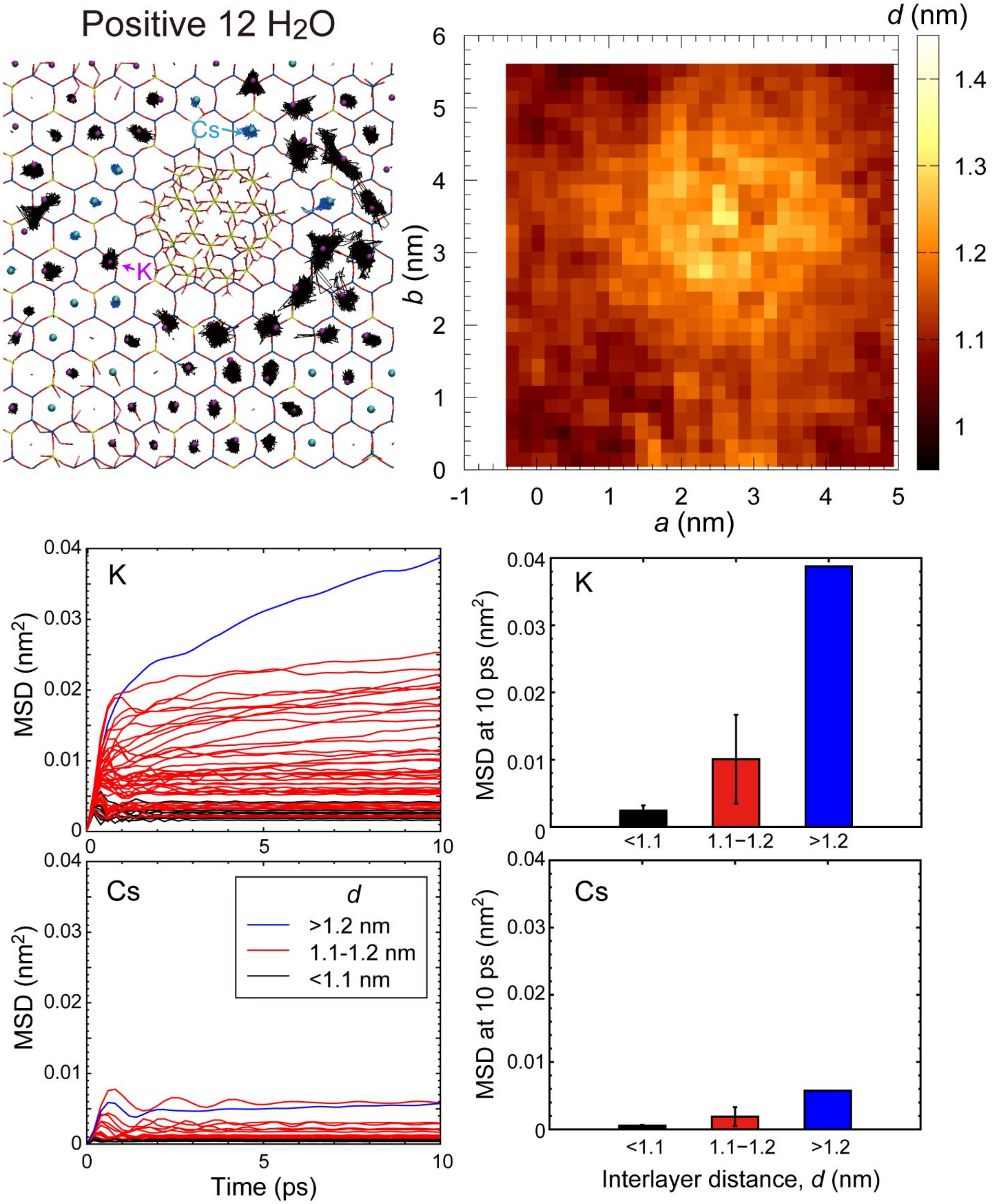

Figure S3. (Top left) Trajectories of interlayer Cs (blue lines) and K ions (black lines) near the Positively charged hydroxy Al ($Al_{24}(OH)_{60}^{12+}$ • $12H_2O$) region. (Top right) Their interlayer distances. (Bottom left) Mean square displacement (MSD) of ions. The color indicates the ranges

of interlayer distances where ions are positioned. (Bottom right) The average and standard deviations of MSD at 10 ps.

DFT Calculations on Dehydrated Models

To assess the thermodynamic feasibility of dehydration during Cs^+ adsorption, we performed additional DFT calculations using dehydrated gibbsite cluster models. For each hydrated model (Edge, Inside, and On-Top), potential dehydration sites were systematically explored by removing different water molecules. To maintain structural relevance, dehydration candidates were limited to water molecules formed by two structural hydroxyl groups within the gibbsite octahedral framework. Water molecules added at the cluster edges to maintain the coordination number of Al atoms were excluded. The dehydration energy (ΔE_{dehyd}) was calculated as:

$$\Delta E_{\text{dehyd}} = E_{\text{dehyd}} + E_{\text{H2O}} - E_{\text{hyd}}$$

where E_{H2O} is the energy of a single water molecule in SMD (Water) solvent (−76.41085947 Hartree) and E_{hyd} refers to the energy of the original Cs-adsorbed gibbsite model without dehydration (shown in Figure 12). The most stable dehydrated configurations identified are summarized in Table S4, and their structures are shown in Figure S4.

Table S4. Calculated total energies and dehydration energies (ΔE_{dehyd}) for hydrated (original Cs^+–Gibbsite) and dehydrated models.

Cs site	E_{hyd} (Ha)	$E_{\text{dehyd}} + E_{\text{H2O}}$ (Ha)	ΔE_{dehyd} (kJ/mol)
(a) Edge	−5331.297431	−5331.251003	+121.89
(b) On-Top	−5331.273043	−5331.271638	+3.69
(c) Inside	−5331.282347	−5331.240127	+110.84

All dehydrated models exhibit positive dehydration energies (ΔE_{dehyd}), indicating that dehydration is thermodynamically unfavorable under the studied conditions. While the On-Top site shows a relatively smaller energy penalty (+3.69 kJ/mol) compared to the Edge and Inside sites (>110 kJ/mol), this small value would primarily influence the population ratio between hydrated and dehydrated On-Top configurations.

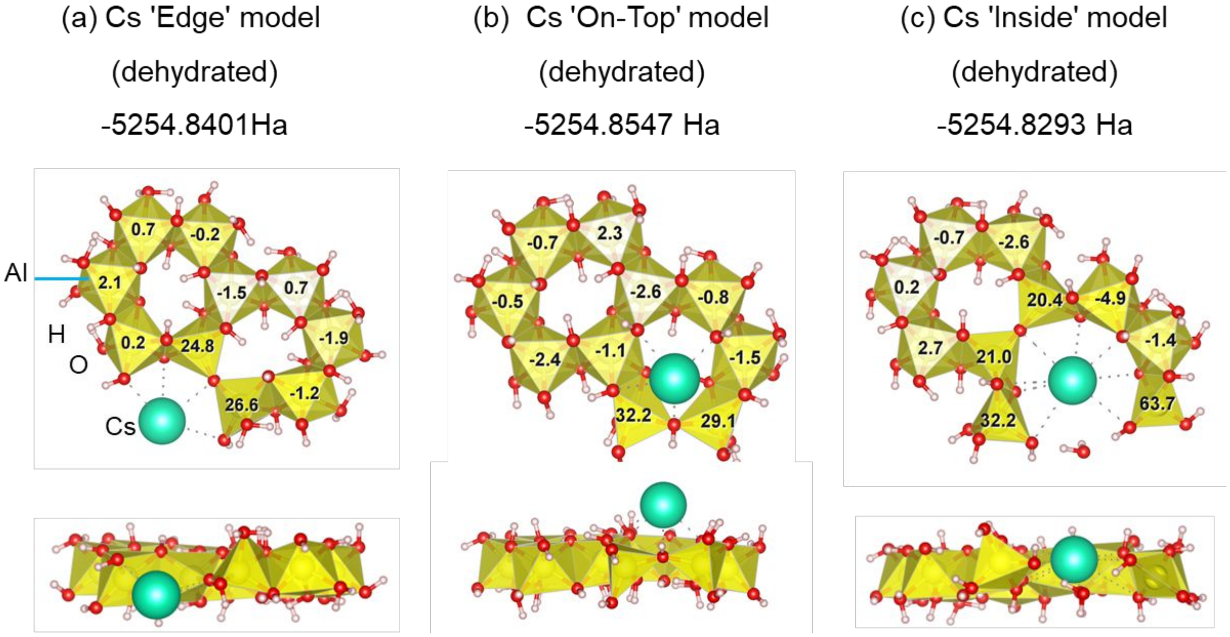

Figure S4. Optimized structures and calculated ^{27}Al NMR chemical shifts for dehydrated gibbsite clusters with Cs^+ ($CsAl_{10}O_{37}H_{43}$): (a) '*Edge*' adsorption, (b) '*On-Top*' adsorption, and (c) '*Inside*' fixation site models. To investigate structural dehydration, these models were obtained by removing a water molecule formed from two structural hydroxyl groups within the $6AlO_6$ ring from the presented configurations shown in Figure 12, while keeping the neutralizing water molecules at the edges intact. Numbers indicate NMR chemical shifts relative to the bare model (Figure 12(a)) average (557.6 ppm) for ^{27}Al. Note that while these dehydrated configurations seem to reproduce the experimental $^{[5]}$Al NMR signals (20-35 ppm), all these dehydrated configurations are energetically unfavorable compared to the hydrated states (see Table S4). Level of theory: ωB97X-D4.rev/def2-TZVPD with SMD (Water).

REFERENCES

(1) Sakuma, H.; Kawamura, K. Structure and Dynamics of Water on Muscovite Mica Surfaces. *Geochim. Cosmochim. Acta* **2009**, *73* (14), 4100–4110.

(2) Sakuma, H.; Kawamura, K. Structure and Dynamics of Water on Li^+-, Na^+-, K^+-, Cs^+-, H_3O^+-Exchanged Muscovite Surfaces: A Molecular Dynamics Study. *Geochim. Cosmochim. Acta* **2011**, *75* (1), 63–81.

(3) Sakuma, H.; Kondo, T.; Nakao, H.; Shiraki, K.; Kawamura, K. Structure of Hydrated Sodium Ions and Water Molecules Adsorbed on the Mica/Water Interface. *J. Phys. Chem. C* **2011**, *115* (32), 15959–15964.

(4) Itoh, H.; Sakuma, H. Dielectric Constant of Water as a Function of Separation in a Slab Geometry: A Molecular Dynamics Study. *J. Chem. Phys.* **2015**, *142* (18).

(5) Sakuma, H.; Ichiki, M. Electrical Conductivity of NaCl-H_2O Fluid in the Crust. *J. Geophys. Res. Solid Earth* **2016**, *121* (2), 577–594.

(6) Sakuma, H.; Ichiki, M.; Kawamura, K.; Fuji-ta, K. Prediction of Physical Properties of Water under Extremely Supercritical Conditions: A Molecular Dynamics Study. *J. Chem. Phys.* **2013**, *138* (13), 134506.

(7) Kitamura, K.; Sakuma, H.; Nishizawa, O. Effect of Temperature on Elastic Properties of Biotite and Biotite-Rich Rocks: Estimation from Experiment and Molecular Dynamics Simulation. *Geophys. J. Int.* **2022**, *231*, 269–289.

(8) Kresse, G.; Furthmüller, J. Efficient Iterative Schemes for Ab Initio Total-Energy Calculations Using a Plane-Wave Basis Set. *Phys. Rev. B Condens. Matter* **1996**, *54* (16), 11169–11186.

(9) Kresse, G.; Joubert, D. From Ultrasoft Pseudopotentials to the Projector Augmented-Wave Method. *Phys. Rev. B Condens. Matter* **1999**, *59* (3), 1758–1775.

(10) Pickard, C. J.; Mauri, F. All-Electron Magnetic Response with Pseudopotentials: NMR Chemical Shifts. *Physical Review B* **2001**, 63, 245101.
